# Implementing adolescent SBIRT in an urban federally qualified health center: generalist vs. specialist service delivery models

**DOI:** 10.1186/1940-0640-10-S2-O24

**Published:** 2015-09-24

**Authors:** Shannon Gwin Mitchell, Arethusa S Kirk, Marla Oros, Jan Gryczynski, Kristi Dusek, Colleen Hosler, Robert P Schwartz, Barry S Brown, Carolina Barbosa, Laura J Dunlap, David W Lounsbury, Kevin E O'Grady

**Affiliations:** 1 Friends Research Institute, Baltimore, USA; 2 Total Health Care, Baltimore, USA; 3 Mosaic Group, Baltimore, USA; 4 Friends Research Institute, Baltimore, USA; 5 Friends Research Institute, Baltimore, USA; 6 Mosaic Group, Baltimore, USA; 7 Friends Research Institute, Baltimore, USA; 8 University of North Carolina at Wilmington, Wilmington, USA; 9 RTI International, Chicago, USA; 10 RTI International, Research Triangle Park, USA; 11 Yeshiva University, Bronx, USA; 12 University of Maryland, College Park, USA

## Background

Little is known about how best to implement SBIRT services in pediatric health care settings or who, optimally, should provide brief interventions when on-site behavioral health is available. The objective of this presentation is to present results from a cluster randomized trial examining implementation of adolescent SBIRT services for substance use within a US federally qualified healthcare system. Two different implementation models for conducting brief interventions (BIs) were compared using randomization at the clinic level to either: the Generalist Model (BI provided by primary care provider) or the Specialist Model (BI provided by behavioral health specialist).

## Material and methods

Multilevel logistic regression modeling was used to examine differences by Condition in rates of successful delivery and documentation of the following services: (a) screening (of all adolescent patients ages 12-17), (b) brief advice (for patients reporting alcohol or drug use but scoring ≥2 on the CRAFFT), and (c) brief intervention (patients scoring <2 on CRAFFT, delivered using either the Specialist or Generalist models). Due to the organization transitioning to a new electronic medical record (EMR) in month 6 of the study, data on BA and BI are currently limited to extractions from the new EMR.

## Results

Multilevel logistic regression analyses taking into account the cluster-randomized design showed no significant differences between Generalist and Specialist conditions in rates of screening (OR=1.27; p=.55), with significant volatility over time (<.001) and variation by sites. In the post-EMR transition, Generalist sites were not significantly more likely to deliver appropriate BA (OR=1.34; p=.70) or BI (OR=1.53; p=.36) than Specialist sites. Site-level intraclass correlations were higher than anticipated. Future analyses will examine practices for the full implementation period and subsequent to the removal of implementation support resources.

## Conclusions

Both service delivery models showed promise for delivering BIs but the high rates of variability within sites demonstrate a need for further examination.

